# Introgression and gene family contraction drive the evolution of lifestyle and host shifts of hypocrealean fungi

**DOI:** 10.1080/21501203.2018.1478333

**Published:** 2018-05-24

**Authors:** Weiwei Zhang, Xiaoling Zhang, Kuan Li, Chengshu Wang, Lei Cai, Wenying Zhuang, Meichun Xiang, Xingzhong Liu

**Affiliations:** a State Key Laboratory of Mycology, Institute of Microbiology, Chinese Academy of Sciences, Beijing, China; b University of Chinese Academy of Sciences, Beijing, China; c Key Laboratory of Insect Developmental and Evolutionary Biology, Institute of Plant Physiology and Ecology, Shanghai Institutes for Biological Sciences, Chinese Academy of Sciences, Shanghai, China

**Keywords:** Phylogenomic, evolutionary process, introgression, gene contraction, host shift

## Abstract

Hypocrealean fungi (Ascomycota) are known for their diversity of lifestyles. Their vital influences on agricultural and natural ecosystems have resulted in a number of sequenced genomes, which provide essential data for genomic analysis. Totally, 45 hypocrealean fungal genomes constructed a phylogeny. The phylogeny showed that plant pathogens in Nectriaceae diverged earliest, followed by animal pathogens in Cordycipitaceae, Ophiocordycipitaceae and Clavicipitaceae with mycoparasites in Hypocreaceae. Insect/nematode pathogens and grass endophytes in Clavicipitaceae diverged at last. Gene families associated with host-derived nutrients are significantly contracted in diverged lineages compared with the ancestral species. Introgression was detected in certain lineages of hypocrealean fungi, and the main functions of the genes located in the introgressed regions are involved in host recognition, transcriptional regulation, stress response and cell growth regulation. These results indicate that contraction of gene families and introgression might be main mechanisms to drive lifestyle differentiation and evolution and host shift of hypocrealean fungi.

## 1. Introduction

Hypocreales (Ascomycota) containing nine recognised families and over 2600 species (Rogerson 1970; Kirk et al. 2008) is one of the most important orders in Ascomycota. Species within hypocreales have evolved various lifestyles including saprophytism, endophytism and parasitism on plants, insects, nematodes and other fungi (Berbee 2001). The evolution of plant and animal pathogens and the origin of the grass endophytes from insect pathogens in Clavicipitaceae were documented by multigene phylogenetic analysis (Spatafora et al. 2007; Sung et al. 2008). Generally, different families display distinct host associations. Nectriaceae includes numerous important plant pathogens such as *Fusarium* that cause serious plant diseases and economical losses (De Wolf et al. 2003; Summerell et al. 2011). Cordycipitaceae represented by *Cordyceps* spp. includes well-known insect pathogens and medicinal fungi (Sung et al. 2007; Zheng et al. 2011). Ophiocordycipitaceae also includes a large number of insect and nematode pathogens and medicinal fungi, such as *Ophiocordyceps sinensis, Hirsutella minnesotensis* and *Hirsutella rhossiliensis* (Jaffee and Zehr 1982; Chen et al. 2000). Meanwhile, Clavicipitaceae is composed of grass endophytes that benefit plants but impair grass-feeding animals (Clay 1988; White et al. 2003), as well as insect and nematode pathogens such as the *Metarhizium* spp. and *Pochonia* spp.

Vital impacts of hypocrealean fungi on agriculture, ecosystems and human life have led to a number of genomes being sequenced (Rogerson 1970). Most of the research on genomics in Hypocreales are mainly focused on gene function related to phylogeny, development and pathogenesis, and has revealed the sophisticated strategies associated with the adaption to various lifestyles (Klosterman et al. 2011; Rouxel et al. 2011). The subtilisins and chitinases, for example, have been shown to be involved in the pathogenesis of insects (Gao et al. 2011). Polysaccharide lyases (PLs) and glycoside hydrolases (GHs), enzymes involved in the breakdown of pectin and cellulose in plant cell walls play a role in the infection of plants (Klosterman et al. 2011). However, the evolutionary history and potential mechanisms of lifestyle changes are not comprehensively understood at the genomic level. The availability of numerous genome sequences of hypocrealean fungi provides an opportunity to examine evolutionary mechanisms in Hypocreales.

Hybridisation and interbreeding between species can lead to adaptive introgression by transmitting beneficial alleles and has the potential to influence adaptation and speciation in a variety of ways, which can happen during either sympatric speciation or the secondary contact phase of allopatric speciation (Arnold 2004). Recent studies have revealed several mechanisms, including introgression, that can lead to adaptive divergence, especially in rapidly radiating groups (Pease et al. 2016). There are numerous striking examples of adaptive introgression in plants, animals and humans that illustrate functional introgressed loci contributing to ecologically and reproductively significant traits (Whitney et al. 2006; Song et al. 2011; Sankararaman et al. 2014; Lamichhaney et al. 2015). *Epichloë* spp., grass endophytes in Hypocreales, contain many species that have evolve through a complex process of hybridisation (Moon et al. 2004), indicating there is high possibility for the occurrence of introgression.

In order to study the evolutionary history and mechanism of cross-kingdom host adaptation of fungi in Hypocreales, a total of 45 sequenced genomes were selected, including two newly sequenced genomes, e.g. the nematode endoparasite *Hirsutella rhossiliensis* (Table S1) sequenced for this project and the pathogenic fungus *Clonostachys rosea* (Toledo et al. 2006; Zhang et al. 2008). The purposes of this study are to characterise the evolutionary patterns of lifestyle shifts in Hypocreales and to illustrate mechanisms that drive the evolution of diverse lifestyles and host shift.

## 2. Materials and method

### 2.1. Fungal strains and genome sequencing

*Hirsutella rhossiliensis* is a dominant parasite of juveniles of the soybean cyst nematode (SCN), *Heterodera glycines*) (Liu and Chen 2000). Strain OWVT-1 was isolated from SCN juvenile in Minnesota, USA and has shown biocontrol potential against nematodes. A single spore isolate of OWVT-1 was cultured on potato dextrose agar (PDA, BD^TM^, New Jersey, U.S.A.) plate for 4 weeks and the mycelium was harvested for genomic DNA preparation using the CTAB/SDS/Proteinase K method (Möller et al. 1992). Whole-genome shotgun sequencing of OWVT-1 was performed using Illumina next generation sequencing technology. DNA libraries with 170, 500, 2 and 5 kb inserts were constructed and sequenced with an Illumina Genome Analyzer at the Beijing Genomics Institute (BGI, Shenzhen, China). The genome was sequenced to approximately 128-fold coverage and assembled using SOAP denovo (Li et al. 2009). Assembly yielded 3543 scaffolds and a genome size of 50.39 Mb.

### 2.2. Gene prediction and annotation

To accurately compare the gene numbers and gene distribution in each fungus, the gene structures were predicted for *H. rhossiliensis* as well as for all other fungi with the same algorithms, Evidence Modeler (Haas et al. 2008), using *Fusarium graminearum* sequence as a reference. Finally, functional predictions were performed by BLASTX search against a protein database and InterProscan searches against protein domain databases (Zdobnov and Apweiler 2001).

### 2.3. Orthology and phylogenomic analysis

Using HMM models, putative orthologs were identified against the BUSCO (Benchmarking sets of Universal Single-Copy Orthologs) database, as the highest full sequence HMM bit score with a minimum *E*-value of e^−50^. Total 627 orthologous proteins were extracted and aligned with MAFFT (Kazutaka and Standley 2013). The program RAxML was used to create a maximum likelihood tree (Stamatakis 2006). The estimation of the evolution of fungal life-strategies was performed using RASP based on the results of RAxML (Yu et al. 2015).

The program BEAST v1.7.4 was used to estimate the divergence time between the compared species, using the orthologous protein sequences (Drummond et al. 2012). The maximum likelihood tree constructed in RAxML (above) was used as the phylogeny. The calibration point of the origin of Ascomycota estimated at 600 Ma was used to estimate divergence times as soft constraints following a uniform limitation (Lücking et al. 2009).

### 2.4. Protein family classification and repeat analysis

Protein families of the whole genomes were classified by analysis of genes descended from a common ancestor. BLAST searching against the FUNCAT database and PHI (pathogen–host interaction) provided a global view of the gene functions (Ruepp et al. 2004; Winnenburg et al. 2006). Statistics for the gene abundance were performed by *t*-test and was corrected by false discovery rate (FDR) test with the *p* < 0.01. Putative enzymes involved in carbohydrate utilisation were identified by BLAST searching against a carbohydrate-active enzymes database (CAZymes) (http://www.cazy.org/). Putative protease families were classified by BLAST against the MEROPS database. Fungal secondary metabolite pathways were analysed with the program SMURF (http://www.jcvi.org/smurf/index.php). The evolution of the protein family sizes and expansion and contractions were analysed by CAFE (De Bie et al. 2006). Simple repeat and transposable elements (TEs) were annotated using BLAST against the RepeatMasker library (http://www.repeatmasker.org/) (Tempel 2012).

### 2.5. Testing for introgression and host-specific evolution

Single nucleotide polymorphisms (SNPs) were detected globally by MUMmer and combined using VCFtools (Kurtz et al. 2004). The *D*-statistic was used to test the phylogenetic distribution of SNPs that display either an ABBA or BABA allelic configuration. *D*-statistic was calculated with (Pease and Rosenzweig 2015):

$$D\left(P_{1},P_{2},P_{3},O\right)=\;\frac{\sum C_{ABAB}\left(i\right)-\;\sum C_{BABA}\left(i\right)}{\sum C_{ABAB}\left(i\right)+\;\sum C_{BABA}\left(i\right)},$$

using window sizes of 5, 50 and 100 kb where *C*_ABBA_(*i*) and *C*_ABAB_(*i*) are counts of whether or not specified pattern (ABBA or BABA) at the *i*th site in the genome was observed and were calculated using the following equation:

$$C_{ABAB}\left(i\right)=\left(1-{\hat{p}}_{i1}\right){\hat{p}}_{i2}{\hat{p}}_{i3}\left(1-{\hat{p}}_{i4}\right)$$

$$C_{BABA}\left(i\right)={\hat{p}}_{i1}\left(1-{\hat{p}}_{i2}\right){\hat{p}}_{i3}\left(1-{\hat{p}}_{i4}\right)$$

Under the null hypothesis of no introgression, *D* approached zero and the candidate loci were assessed for significance using a *z*-test with *p* < 0.01. To confirm candidate introgression loci, *f_d_* was calculated using the following equation:

$$f_{d}=\frac{S\left(P_{1},P_{2},P_{3},O\right)}{S\left(P_{1},P_{D},P_{D},O\right)}$$

where among the compared the four taxa of *P*_1_, *P*_2_, *P*_3_ and *O, P_D_* can be either *P*_2_ or *P*_3_, which has higher frequency of the derived allele. We excluded the windows with *f_d_* lower than the top 10% *f_d_* estimators to reduce the bias of heterogeneity in genetic variation. To rule out false positive introgression loci due to incomplete lineage sorting, mean DNA sequence divergence (*d_xy_*) was calculated and compared between the candidate loci with the whole scaffold regions using the following equation:

$$d_{xy}=\frac{1}{n}\overset{i=1}{\underset{n}{\sum }}\left({\hat{p}}_{ix}\left(1-{\hat{p}}_{iy}\right)+{\hat{p}}_{iy}\left(1-{\hat{p}}_{ix}\right)\right),$$

where *p_x_* and *p_y_* refer to reference allele frequency in taxa *x* and *y*. Standard error was calculated for the windows in each chromosome. The two values were compared using a *z*-test with *p* < 0.01.

### 2.6. Annotation of introgression loci

To investigate the functions of introgressed loci, the genes associated with the windows with significant introgression signal were identified. Genes that cover at least one introgressed locus were extracted. Functions of these genes were annotated by blasting against PFAM, FUNCAT and GO database (Harris et al. 2004; Ruepp et al. 2004; Marco et al. 2012).

## 3. Results

### 3.1. Phylogeny and host shifts of hypocrealean fungi

**Figure 1.**
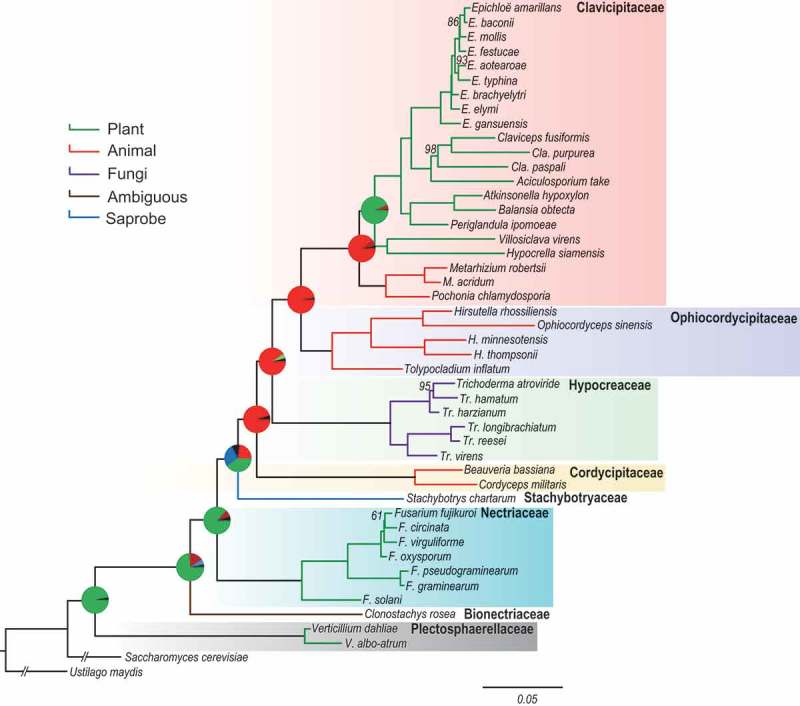
Phylogenetic relationships of the species of the order Hypocreales. Bootstrap values are 100, except the marked nodes, and provide evidence for the tree structure. Pie charts at each nodes show the possibility of the ancestral life strategies with different colours.

Phylogenomic analysis of 45 fungal genomes (Table 1) from seven families in Hypocreales was conducted using *Ustilago maydis* (Basidiomycota) and *Saccharomyces cerevisiae* (Ascomycota) as an outgroup (Rogerson 1970). A dataset comprised of 627 genes encoding single-copy homologous proteins obtained by blasting against the BUSCO database was used to construct the phylogenetic relationships using RAxML (Figure 1) (Alexandros 2014). Using the divergence time for Ascomycota at 600 million years ago (Mya) (Lücking et al. 2009) for calibration, the divergence time for Hypocreales was estimated to be 217 Mya by BEAST (Figure S1). The resulting phylogeny had high boot-strap support for all families and was mostly consistent with results of a previous multilocus phylogeny (Spatafora et al. 2007). *Verticillium* spp. in Plectospharellaceae, highly virulent plant pathogens, were the earliest diverging lineage in the Hypocreales. *Clonostachys rosea* in Bionectriaceae evolved to infect various hosts including animals, plants, and other fungi and subsequently the families of hypocrealean fungi diverged to various lifestyles. *Fusarium* spp., as representatives of Nectriaceae, developed after Bionectriaceae to be plant pathogens or weak insect pathogens. In turn, several monophyletic lineages corresponding to Cordycipitaceae, Ophiocordycipitaceae and Clavicipitaceae diverged to insect and nematode pathogens. The mycoparasitic *Trichoderma* spp. in Hypocreaceae were nested within the animal pathogenic lineages between Cordycipitaceae and Ophiocordycipitaceae As one of the most diverged lineages, fungi in Clavicipitaceae split into two clades corresponding to insect pathogens and grass endophytes. Available data suggest that early diverging insect pathogenic lineages within Clavicipitaceae originated from other insect pathogen lineages such as Ophiocordycipitaceae and then reverted to a plant host as grass endophytes (Figure 1). Divergence of Clavicipitalean endophyte at 70 Mya was consistent with the divergence time of their plant hosts in Gramineae (Paterson et al. 2004), indicating that the grass endophytes may represent a rapid radiation resulting from adaption to and coevolution with the plant hosts.

**Table 1.** Species and accession numbers of the genomic data used for phylogenomic analysis.

Families	Species	Accession numbers
Clavicipitaceae	*Aciculosporium take*	AFQZ00000000
Clavicipitaceae	*Atkinsonella hypoxylon*	JFHB00000000
Clavicipitaceae	*Balansia obtecta*	JFZS00000000
Cordycipitaceae	*Beauveria bassiana*	ADAH00000000
Clavicipitaceae	*Claviceps fusiformis*	AFRA00000000
Clavicipitaceae	*Claviceps paspali*	AFRC00000000
Clavicipitaceae	*Claviceps purpurea*	CAGA00000000
Bionectriaceae	*Clonostachys rosea*	JYFM00000000
Cordycipitaceae	*Cordyceps militaris*	AEVU00000000
Clavicipitaceae	*Epichloe amarillans*	AFRF00000000
Clavicipitaceae	*Epichloe aotearoae*	JFGX00000000
Clavicipitaceae	*Epichloe baconii*	JFGY00000000
Clavicipitaceae	*Epichloe brachyelytri*	AFRB00000000
Clavicipitaceae	*Epichloe elymi*	AMDJ00000000
Clavicipitaceae	*Epichloe festucae*	AFRX00000000
Clavicipitaceae	*Epichloe gansuensis*	AFRE00000000
Clavicipitaceae	*Epichloe mollis*	JFGW00000000
Clavicipitaceae	*Epichloe typhina*	AMDI00000000
Nectriaceae	*Fusarium circinata*	JRVE00000000
Nectriaceae	*Fusarium fujikuroi*	JRVG00000000
Hypocreaceae	*Fusarium graminearum*	AACM00000000
Nectriaceae	*Fusarium oxysporum*	AAXH00000000
Nectriaceae	*Fusarium pseudograminearum*	AFNW00000000
Nectriaceae	*Fusarium solani*	ACJF00000000
Nectriaceae	*Fusarium virguliforme*	AEYB00000000
Ophiocordycipitaceae	*Hirsutella minnesotensis*	JPUM00000000
Ophiocordycipitaceae	*Hirsutella rhossiliensis*	MPJM00000000
Ophiocordycipitaceae	*Hirsutella thompsonii*	APKU00000000
Clavicipitaceae	*Hypocrella siamensis*	JMQE00000000
Clavicipitaceae	*Metarhizium acridum*	ADNI00000000
Clavicipitaceae	*Metarhizium anisopliae*	AZNF00000000
Ophiocordycipitaceae	*Ophiocordyceps sinensis*	ANOV00000000
Clavicipitaceae	*Periglandula ipomoeae*	AFRD00000000
Clavicipitaceae	*Pochonia chlamydosporia*	AOSW00000000
Stachbotryaceae	*Stachybotrys chartarum*	LDEE00000000
Ophiocordycipitaceae	*Tolypocladium inflatum*	AOHE00000000
Hypocreaceae	*Trichoderma atroviride*	JZUQ00000000
Hypocreaceae	*Trichoderma hamatum*	ANCB00000000
Hypocreaceae	*Trichoderma harzianum*	JNNP00000000
Hypocreaceae	*Trichoderma longibrachiatum*	ANBJ00000000
Hypocreaceae	*Trichoderma reesei*	AAIL00000000
Hypocreaceae	*Trichoderma virens*	ABDF00000000
Incertae sedis	*Verticillium albo-atrum*	ABPE00000000
Incertae sedis	*Verticillium dahiliae*	ABJE00000000
Clavicipitaceae	*Villosiclava virens*	JHTR00000000

The ancestral host-associations and nutrient requirements at the nodes of each lineage were obtained using the program RASP (Figure 1, Figure S2) (Spatafora and Bushley 2015). Results suggested that the fungi in early diverging nodes mainly utilised plant-based nutrients as pathogens and subsequently shifted to simpler nutrient resources including insect-, fungi- and nematode-based resources, and finally a reversal to plant-based nutrition as symbionts (Yu et al. 2015). The most recently diverged lineage, Clavicipitaceae, might also originate from insect/nematode pathogens from other families (probability = 0.9688). Insect/nematode pathogens in Clavicipitaceae most likely evolved from other insect pathogenic families and then reverted to plant hosts as grass endophytes (probability = 0.8893). This evolutionary scenario is also supported by the shared secondary metabolism associated with animal-toxins in insect/nematode pathogens and endophytes in Clavicipitaceae (Spatafora and Bushley 2015).

### 3.2. Genome characteristics and lifestyles

**Figure 2.**
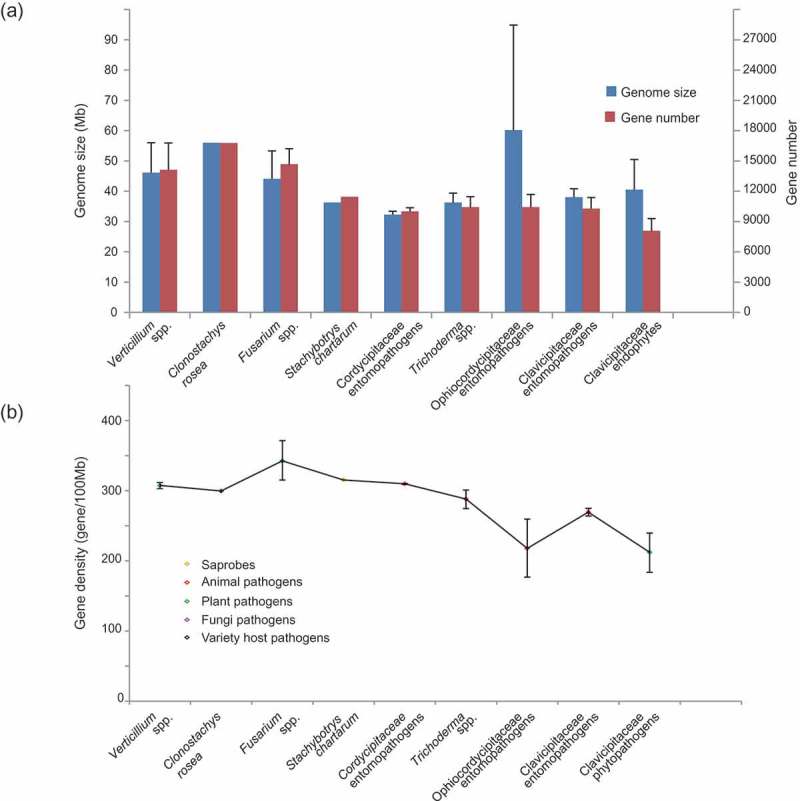
Average genome sizes, gene numbers and gene densities of fungal lineages employing different life strategies. (A) Left axis shows genome sizes (Mb) and right axis shows gene numbers. Fungal lineages are classified by the same life strategies. (B) Gene densities of fungal lineages employing different life strategies.

Genome size expansions and low gene density can result from the accumulation of repetitive sequences (Wang and St Leger 2013). The genome sizes, gene numbers and gene densities were compared for the 45 fungal genomes. Genome size ranged from the largest, an average of 60.2 Mb for Ophiocordycipitaceae, to the smallest, an average of 32.3 Mb for Clavicipitaceae (Figure 2, Table S2). The largest number of genes were 16,779 predicted in the genome of *Clo. rosea* which interacts with diverse hosts (Figure 2(a)), while the smallest number of genes was 7480 predicted in the genome of *Epichloë* spp. that live as symbiotic endophytes in grasses (Table S2). Low gene density was found in host-specific fungi in both Ophiocordycipitaceae parasitizing animals and Clavicipitaceae colonising plants as symbiotic endophytes (Figure 2(b)). On the other hand, the contents of repetitive sequences and TEs were detected in both grass endophytes *Epichloë* spp., an average of 46% TEs, and Ophiocordycipitaceae with an average of 31%. Although the plant parasitic *Claviceps* spp. are phylogenetically close to *Epichloë* spp., they contained only 13% TEs. The nematode endoparasitic *Hirsutella* spp. and ghost moths parasite *O. sinensis* are both host density-dependent obligate pathogens, indicated their strong interactions with and dependence upon their hosts (Hu et al. 2013; Lai et al. 2014). The large number of TEs in the genomes of these fungi might be associated with their obligate interactions with their hosts.

**Figure 3.**
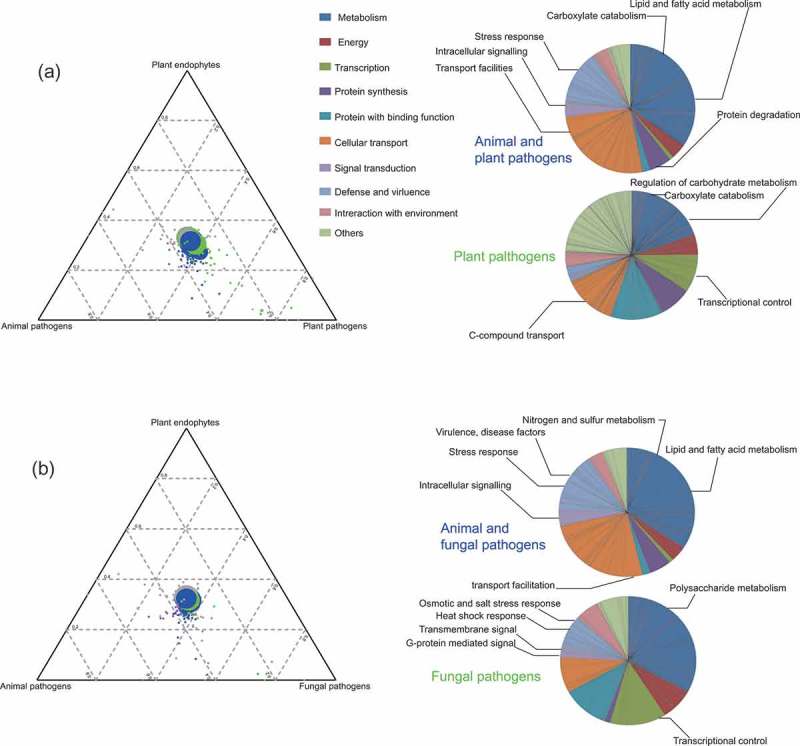
Significant expansion of gene families base on FUNCAT database compared with grass endophytes. The significance was tested by FDR < 0.05. (A) The expanded gene families shared by plant pathogens and animal pathogens were represented with blue circles in the triangle; the expanded gene families of plant pathogens were coloured green; the expanded gene families of animal pathogens were coloured red; gene families without significance were coloured grey; detailed gene families were represented in the pies. (B) The expanded gene families shared by fungal pathogens and animal pathogens coloured blue; the expanded gene families of fungal pathogens were coloured green; the expanded gene families of animal pathogens were coloured red.

The gene families were functionally annotated by blasting against the FUNCAT database and a total of 566 gene families were identified and compared among each group characterised as plant pathogens, animal pathogens, fungal pathogens and grass endophytes. The plant pathogens, as the earliest diverging lineages had the highest number of gene families (332 families) that were mainly associated with carbohydrate, lipid and nitrogen metabolism, facility transportation, intracellular signal transduction and stress response (Figure 3, Table S3). The mycoparasitic *Trichoderma* shared similar functional gene families (175 families) with plant pathogens but contained more families involved in stress response, G-protein signal transduction and virulence. Although the genomes of insect and nematode pathogenic fungi had fewer identified gene families than those of fungal and plant pathogens, gene families associated with metabolism, stress response and transportation were more abundant, indicating that these gene families might be involved in adaption or virulence on insect or nematode hosts.

The virulent factors are a key component in the interactions of fungi with their hosts. By blasting against PHI database, a total 521 PHI gene families were identified (Table S4). A lower number of PHI gene families in symbiotic endophytic fungi was observed, while fungi interacting with multiple hosts, such as *Clo. rosea* and *Fusarium*, have a much higher number of G-protein coupled receptors (PHI:441) (average 43) than plant pathogenic *Verticillium* (average 14) and other pathogens (average 10), indicating that fungi with multiple lifestyles required more virulent factors to adapt to different hosts. Furthermore, the typical plant pathogens had a larger number of pectinases (PHI:179, PHI:180 and PHI:222) (average 12), enzymes involved in degradation of the plant cell wall and middle lamella, while these enzymes are present in lower numbers of absent in the other types of pathogens. The utilisation of pectin is essential for plant parasitism.

### 3.3. Host nutrient-based evolution

**Figure 4.**
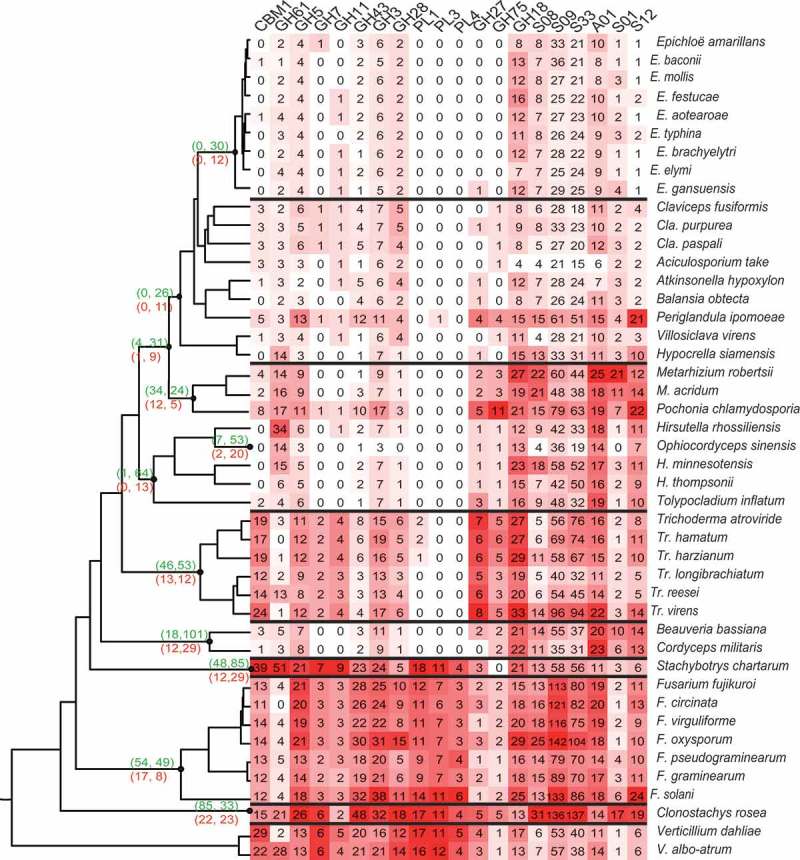
Gene expansion/contraction and gene numbers of corresponding gene families. The numbers of expanded and contracted gene families are shown at the node of divergence by CAFE: upper brackets (expanded families, contracted families) of CAZy database; below brackets (expanded families, contracted families) of MEROPS database.

The utilisation of host-based nutrition is critical for fungal parasitism and includes or carbohydrate-activated enzymes (CAZymes) and as well as proteases, which are among key virulence factors involved in parasitism in various pathogenic fungi (Gao et al. 2011; Klosterman et al. 2011; Xiao et al. 2012). The abundance and types of these enzymes should be key to the adaption to different host-based nutrition. The expansion and contraction of CAZymes and MEROPS, as key enzymes responsible for substrate utilisation, were detected by the programme CAFÉ (Figure 4, Table S5) (De Bie et al. 2006; Rawlings et al. 2006; Cantarel et al. 2009). One significant gene family contractions occurred during the divergence of animal pathogens in Cordycipitaceae with 101 contracted families of CAZymes and 29 of MEROPS. Similarly, a total of 64 CAZyme families and 13 MEROPS families were found contracted in Ophiocordycipitaceae. Though Clavicipitaceae appears to be a dichotomous group with both animal pathogens and endophytes, gene family contraction also occurs during the divergence of grass endophytes.

The distribution of families was analysed by a heat-map of the most differentially presented gene families (Figure 4). The degradation of plant cells are associated with a series of CAZymes including numerous cellulase encoding genes containing carbohydrate-binding module 1, GH3, GH5, GH7, GH11 and GH61 domains as well as pectinases coding genes containing GH28, GH43, PL1, PL2 and PL4 domains presented in our matrix analysis of gene families. The genes that process the degradation of plant cells are abundant in the ancestral plant pathogenic *Verticillium* spp. and saprophytic *Stachybotrys chartarum* genomes, while protease-coding genes were less abundant. The largest numbers of enzyme families were identified in the genomes of *Fusarium* spp. and *Clo. Rosea*, fungi with multiple life-strategies. After the transition from plant pathogens to animal pathogens, the number of genes responsible for plant degradation sharply decreased and the pectinase PLs almost disappear. On the other hand, the number of protease encoding genes also decreased when the lifestyle changed from insect/nematode pathogens to grass endophytes within Clavicipitaceae (Figure 4, Table S5).

### 3.4. Evidence for introgression among species

Introgression lines and introgression have been documented to be common during rapid speciation and host adaption (Arnold 2004). Although introgression has been rarely investigated in the evolution of fungi (Pease and Rosenzweig 2015; Zhang et al. 2015), molecular phylogenetic analysis has demonstrated that the speciation of *Epichloë* species has often involved a complicated processes of hybridisation (Moon et al. 2004). In addition, the close relationships between grass endophytes and insect pathogens in Clavicipitaceae and our results suggest that they also share similar genomic features. Thus, introgression may contribute to the evolution of host-shifts and diverse lifestyles.

**Figure 5.**
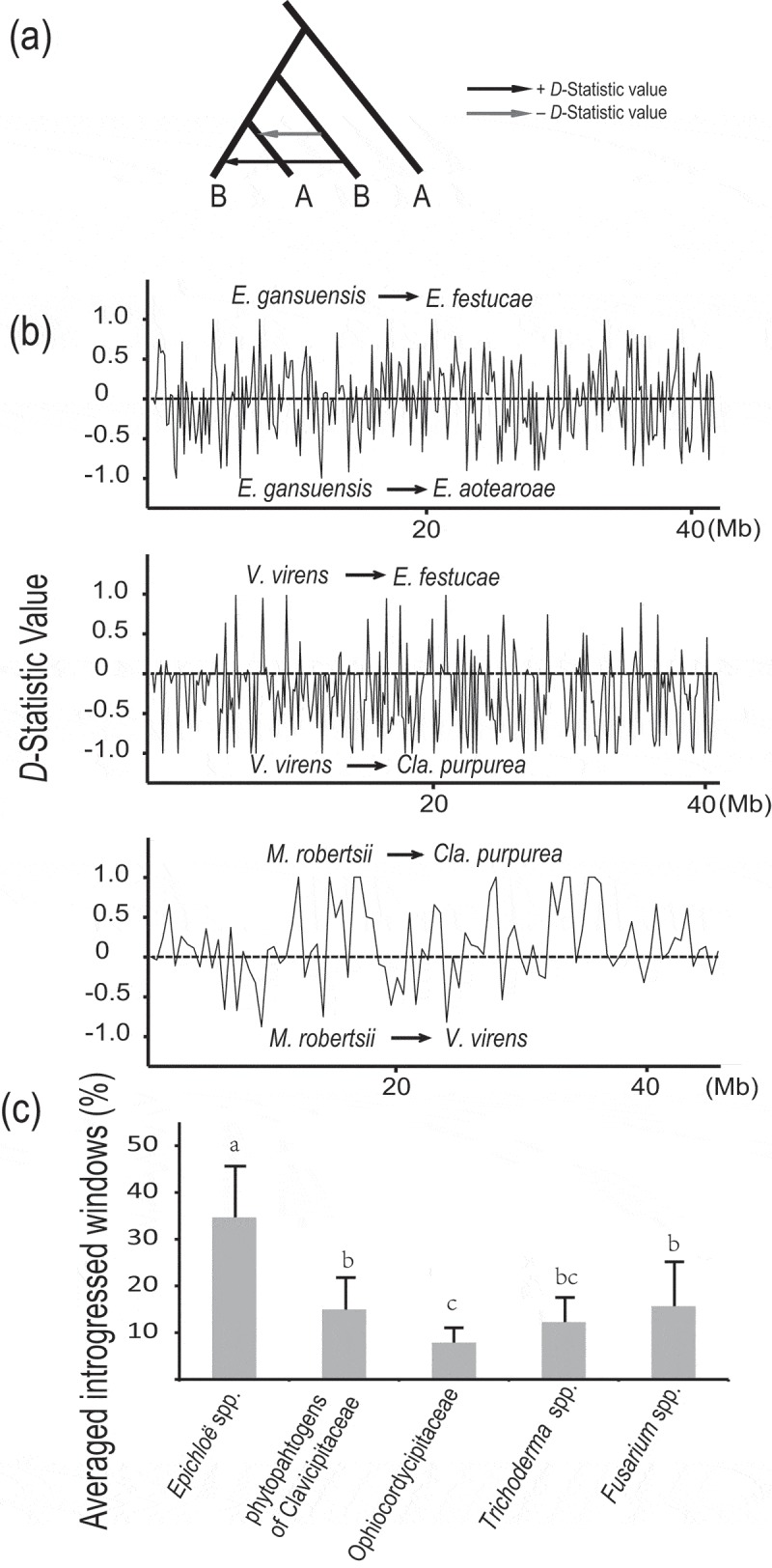
Patterson’s *D*-statistic scans along the whole genome sequences. (A) For the tree topology above, clear introgression patterns are observed. (B) Patterson’s *D*-statistic along the whole genome using 500 kb adjacent windows. (C) Averaged windows with introgressive signal detected by significant *D*-statistic.

Therefore, inter-species introgression in Hypocreales was analysed. A sliding window of 100, 50 and 5 kb was used to compute the *D*-statistic (Hudson 1983) as a signal of introgression between *Epichloë* spp. (*E. festucae, E. aotearoae* and *E. gansuensis*) that originate from sexual hybridisation (Durand et al. 2011). *Claviceps purpurea* was designed as an outgroup. There were 28 among 243 the 100-kb windows detected to be exchanged between *E. gansuensis* and *E. festucae* with significant *D*-statistics through *z*-text (|*D*| > 0.59, *p* < 1 × 10^−3^ and |ABBA − BABA| > 10) (Figure 5(b)). Similarly, 74 out of the 582 50-kb windows and 337 out of the 5816 5-kb windows also had significant *D*-values. These data indicate a high probability of introgression during the speciation of *Epichloë* spp. A 5-kb slide window was also applied to further examine genome-wide introgression and identify the candidate introgressed loci and their functions. The *f*-statistic was calculated for the focal intervals and values significantly lower than 90% *f_d_* were excluded (Pease and Rosenzweig 2015). The DNA sequence divergence (*d_xy_*) was calculated for each candidate introgression interval to distinguish introgression and ancestral variation. There were 59 introgressed 5-kb windows with significantly lower *d_xy_* than that of the whole scaffold region (Table S6). These results reveal a significant signal of introgression between *E. gansuensis* and *E. festucae*, which is to equivalent to the level of introgression observed in distantly related butterfly species (Zhang et al. 2015).

On the other hand, the introgression among the endophytes *E. festucae, Cla. purpurea* and *Villosiclava virens* for which no sexual hybridisation has been reported was examined using the insect pathogenic *Metarhizium robertsii* in Clavicipitaceae as an outgroup. Only 2 out of 253 100-kb windows and 2 out of the 6709 5-kb windows were identified with significant *D* values between *E. festucae* and *Villosiclava virens*, indicating a low possibility for gene introgression (Figure 5(b), Table S6). However, 13 out of 93 100 kb windows and 12 out of the 1842 5-kb windows were identified between the genomes of *Cla. purpurea* and *M. robertsii* (Figure 5(b), Table S6), suggesting that the grass endophytes have genetic resources shared with insect pathogens.

Overall, a coarse inter-subclade frequency of introgression and inferences of the sources of genetic variation were estimated from the *D*-statistics across all the branches within the hypocrealean fungi using 500-kb windows. A total of 100 out of the 112 500-kb windows showed evidence of introgression for at least one clade in the tree (Figure 5(c)). The subclade of grass endophytes in Clavicipitaceae had the largest numbers of windows (average of 9.08%) showing significant inter-species introgression compared with *Trichoderma* spp. (average of 3.59%). Evidence of introgression was also identified between the subclades in Hypocreales. Clavicipitaceae showed approximately 10% of the total number of windows introgressed from Hypocreaceae, Cordycipitaceae and Nectriaceae, respectively. Introgression is often associated with sexual hybridisation during speciation (Geiser et al. 1996; Holder et al. 2001). Hypocreales contains many sexual lineages as teleomorphs for many species are found in nature and the evolution of lifestyle shifts and flexible lifestyle traits could result from introgression during sexual hybridisation. This hypothesis has been proposed previously for Cordycipitaceae (Wang and St Leger 2013). Although our estimates of introgression are based on simple calculations of *D*-statistics, they suggest that hypocrealean fungal species might arise more frequently that previously recognised through sexual hybridisation.

### 3.5. Annotated functions of the introgressed loci

The functions of genes identified within the introgressed 5-kb windows were further investigated. The genes within introgressed regions were extracted and annotated by blasting them against the Pfam and GO databases. Among the total of 34 genes identified as introgressed between *E. gansuensis* and *E. festucae*, genes that encode proteins involved in transcriptional regulation and stress resistance were highly represented among the introgressed genes as well as three genes homologous to CPUR_06320, CPUR_03992 and CPUR_06368 in that are involved in cell growth (Table S7). These genes could be involved in helping these grass endophytes living inside of plant to protect themselves from oxidative and other stresses activated by host defense responses. On the other hand, introgressed genes between *M. robertsii* and *Cla. purpurea* were mainly involved in regulation of metabolism and host recognition. For example, the introgressed THAM_008331, a GTPase-activator protein and THAM_06628, a cAMP dependent protein kinase, highlights the importance of host recognition for these taxa (Table S7).

## 4. Discussion

The 45 genomes included in this study belong to seven out of the nine families currently recognised in Hypocreales. Several species were included in some genera of some families (e.g. *Fusarium, Trichoderma*, etc.). However, due to the limited availability of genomes, only one species in each family of Stachybotryaceae and Bionectriaceae was included in the present analysis, which is less than that of the previous phylogenetic study using multilocus sequencing of ribosomal and protein coding genes (Spatafora et al. 2007). Although insufficient genomic data limited the depth of analysis of some evolution mechanisms such as the host jumping in these fungi (Sung et al. 2007), this study highlights evolutionary processes involved in nutrient-based lifestyles and a comprehensive understanding of the phylogenetic relationships of different lineages. The results suggest that the hypocrealean fungi might originate from a plant-based nutrition. The RASP yielding ancestral lifestyle and the identification of numerous plant parasitism-related genes in the genomes of *Metarhizium* spp. also support this hypothesis (Gao et al. 2011).

To adapt to distinct host-based nutrients, gene families significantly contracted or expanded. Generally, genomic analysis revealed that a higher abundance of CAZymes is associated with the utilisation of plant-based nutrition, while greater numbers of proteases and chitinases are associated with insect-based nutrition, while only proteases are associated with nematode-based nutrition (Dimitrios et al. 2012; Xiao et al. 2012; Lai et al. 2014; Liu et al. 2014). A series of CAZymes involved in carbohydrate degradation were significantly contracted during the lifestyle transition from plant pathogens to other animal pathogenic, mycoparasitic or endophytic lifestyles. Proteases, were mainly associated with animal-based nutrition and may also function as virulence factors, were also significantly contracted during the lifestyle transition from animal pathogens to grass endophytes. Meanwhile, contraction of genes involved in nutrient utilisation might further narrow the host range. Analysis of global distributions of gene functions showed that functions that show a decrease in animal pathogens and grass endophytes are mainly associated with metabolism and nutritional transport. The contraction of G-protein receptors might also limit the host recognition and contribute to limiting the host range. The global contraction of gene families in *O. sinesis* also likely contributes to its narrow host range and obligate parasitism (Hu et al. 2013).

The *D*-statistic test in genomic analysis was first introduced by Green et al. (2010) to evaluate formally whether humans harbour some Neandertal ancestry (Green et al. 2010). Recently, it has been used as a convenient statistic for studying locus-specific introgression of genetic material controlling coloration in *Heliconius butterflies* (Zhang et al. 2015). *D*-statistic analysis requires four species including two sister species, a third species potentially involved in introgression and an outgroup species (Martin et al. 2014). Most investigations of introgression focus on animals and plants, such as horse, butterfly and tomato, that have sexual reproduction during their life-cycles (Pease and Rosenzweig 2015; Zhang et al. 2015). Generally, it is believed that sexual reproduction could lead to a higher occurrence of introgression (Geiser et al. 1996; Holder et al. 2001). However, introgression has rarely been investigated in the evolutionary studies of fungi.

The global analysis of introgression in hypocrealean fungi was conducted and *Clo. Rosea*, an ancestral species that displays diverse life-strategies without significant gene contraction, was used as an outgroup to guarantee the maximum amount of homologous sequences. However, only 40% of the 500-kb windows are homologous to the genome sequences of *Epichloë* spp. that have global gene contractions and large number of TEs. A large number of sequences with a signal of introgression were identified in *Epichloë* spp, supporting previous observations that speciation among *Epichloë* spp. is often associated with sexual reproduction and hybridisation (Moon et al. 2004). Numerous genes are introgressed between *M. robertsi* and *Cla. purpurea*, indicating that the endophytes and animal pathogens share very close ancestors and that speciation in the Clavicipitaceae has involved in frequently introgression. The high frequency of introgression identified among species in Hypocreales provide evidence that adaptive introgression and gene flow among fungi living on similar hosts may contribute to the evolution of the diverse and flexible lifestyles notable for this group of fungi.

In summary, the evolution of distinct host nutrient-based lifestyles of hypocrealean fungi is supported by the contraction and expansion of nutrient utilisation related gene families corresponding to the lifestyle adaptions to various hosts. Plant pathogens appear to be the earliest group from which animal and fungal pathogens evolved, and finally reverted back to a plant-based nutrition as plant endophytes. The observation of global gene family contractions, especially in cellulases encoding genes in the transition from plant pathogens to animal and fungal pathogens, and pectinases encoding genes in the transition from animal pathogens to endophytes. Introgression signals were significantly detected in certain lineages of hypocrealean fungi and the main functions of the genes located in the introgressed regions were related to host recognition, transcriptional regulation, stress response and cell growth regulation. Introgression and gene family contraction/expansion are evolutionary mechanisms that may drive rapid speciation and diverse host shift observed in hypocrealean fungi, one of the most impact group on ecosystem, agriculture and human health.
