# Supplementary figures and images for: Introgression and gene family contraction drive the evolution of lifestyle and host shifts of hypocrealean fungi
Source: Mycology. 2018 May 24;9(3):176–88. doi: 10.1080/21501203.2018.1478333 (PMC6115877; doi:10.1080/21501203.2018.1478333)

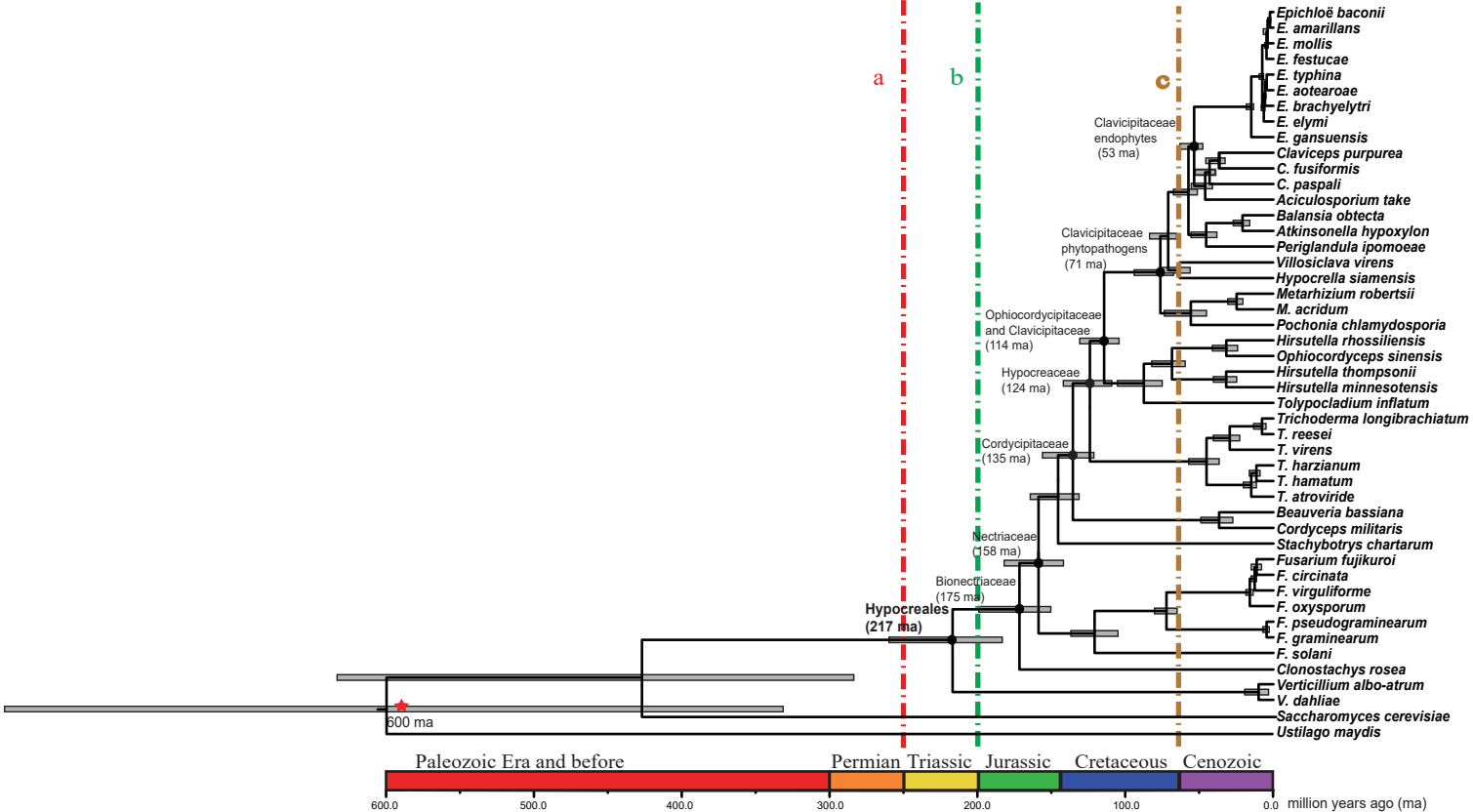

Supplement: Supplemental Material [file TMYC_A_1478333_SM1858.zip › S1_fig.pdf]

Plant pathogen  
 Animal pathogen  
 Fungal pathogen  
 Saprobe

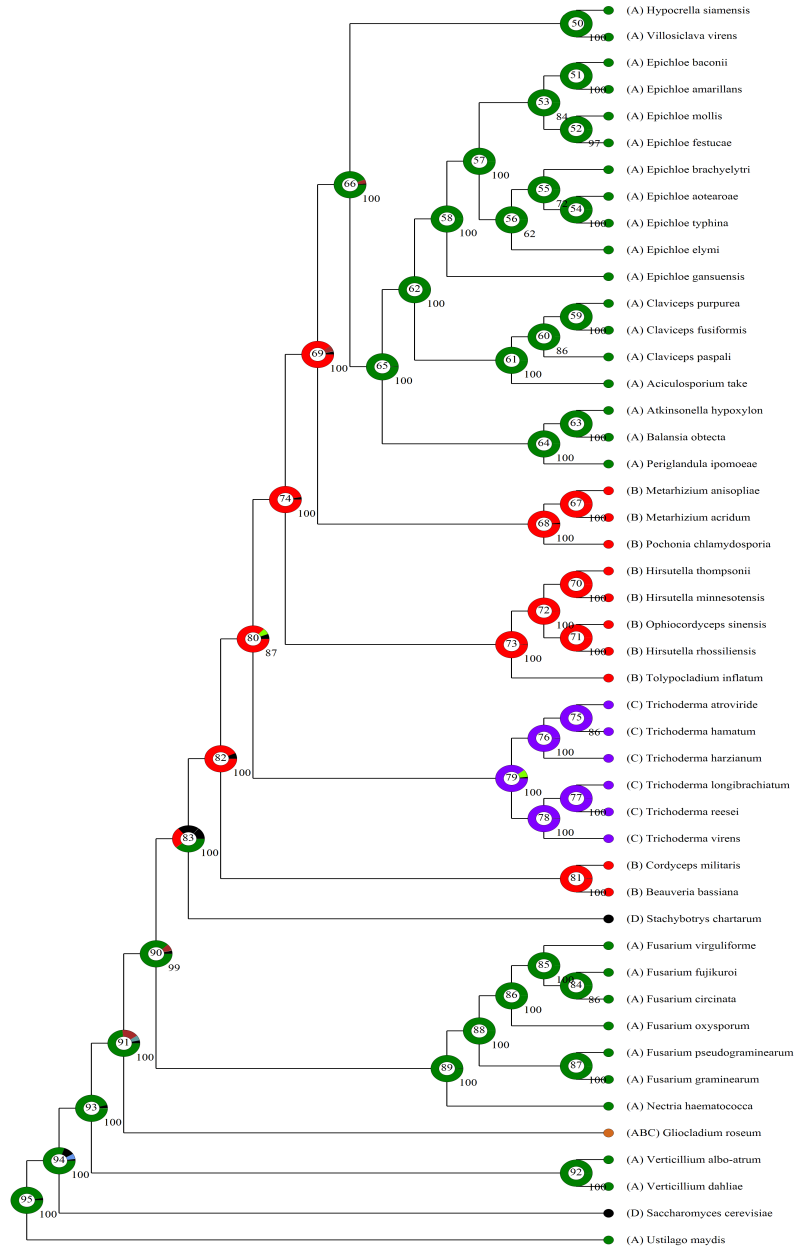

Supplement: Supplemental Material [file TMYC_A_1478333_SM1858.zip › S2_fig.pdf]
